# Fungiform papillae density in patients with burning mouth syndrome and xerostomia

**DOI:** 10.4317/medoral.17611

**Published:** 2011-12-06

**Authors:** Fabio Camacho-Alonso, Pía López-Jornet, Diana Molino-Pagán

**Affiliations:** 1 Full Professor of Oral Medicine. University of Murcia (Spain); 2 Graduated in Dentistry. University of Murcia (Spain)

## Abstract

Objective: The aim of this study was to analyze fungiform papillae density in patients with burning mouth syndrome (BMS) and xerostomia. Study design: In this cross-sectional clinical study, sixty patients were included (20 with BMS, 20 with xerostomia and 20 healthy controls). The fungiform papillae density was analyzed over a small region on the anterior tip of the tongue with the aid of a digital camera. The number of papillae was measured in an area of 19 mm2. Results: The patients with BMS showed significantly higher fungiform papillae density than the patients with xerostomia; though no statistically significant differences were recorded versus the control group. In the BMS group, 65% of all cases presented a density of 71-90 papillae (within an area of 19 mm2), while 10% had more than 90 papillae. On the contrary, 70% of the patients with xerostomia had fewer than 70 papillae in the studied area. Conclusions: The digital camera offers a rapid, noninvasive and relatively simple way to study fungiform papillae density. The patients with BMS have higher fungiform papillae density than the patients with xerostomia.

** Key words:**Tongue, fungiform papillae, burning mouth syndrome, xerostomia.

## Introduction

Burning mouth syndrome (BMS) is a painful condition that occurs mostly in postmenopausal women (1,2). The disease is characterized by unremitting oral burning and/or similar pain, without detectable oral mucosal changes (1-6).

The role of taste sensation in BMS is a complicated issue, though the latest studies point to a possible relationship between taste disorders and the BMS (7-11). The tip of the tongue is usually affected in BMS, and the pain is often accompanied by taste alterations. With regard to gustatory sensitivity in BMS, many authors have reported a decreased ability to taste in about two-thirds of the patients (2-9). Finally, dysgeusia (sometimes a metallic taste) has been described in patients with BMS.

The frequent observation of taste changes and/or sensory/chemosensory dysfunctions in BMS patients has suggested that this syndrome could reflect a neuropathic disorder (10); in particular, peripheral nerve injury has been hypothesized (1,9). Furthermore, some patients with dysgeusia exhibit a loss of inhibitory interactions between the central projection areas of the chorda tympani or glossopharyngeal taste nerves following peripheral injury to either nerve (1,2,12). Grushka and Sessle (9) in a sample of 49 patients with BMS reported disturbances of taste in a 69%. These disturbances were: bitter flavor (33%), metallic (27%) and other combination of taste alterations (10%); nevertheless, these alterations decreased in 60% of the subjects after rinsing with distilled and deionized water. Finally, the authors also observed alterations in the intensity to the taste perception of salt (70% stronger or weaker), sweet (40% weaker), sour (40% stronger) and bitter (35% stronger).

On the other hand, dry mouth due to hyposalivation, is considered to be one of the causes of the atrophy of tongue papilla. In this sense, Yamamoto et al. (13) studied the atrophic of the filiform and fungiform papillae in 44 patients with Sjögren Syndrome (SS), 20 patients with xerostomia and 20 healthy controls. They observed more atrophic papillae in the SS group, although the patients with xerostomia also showed a high degree of atrophy.

The perception of taste is directly dependent upon chemoreceptors on the gustatory papillae located on tongue dorsum. Therefore, the measurement of papillae density can provide information about taste function. In this sense, the aim of this study was to analyze the fungiform papillae density over a small region on the anterior tip of the tongue (with the aid of a digital camera), in patients with BMS and xerostomia.

## Material and Methods

-Study subjects 

A total of 60 patients who went to the Department of Oral Medicine of the Universitary Clinic of Dentistry of the University of Murcia (between january of 2009 and november of 2010) were included in the study (20 with BMS, 20 with xerostomia and 20 healthy controls). The study was approved by the Ethics Committee of the same University.

The information about the aims of this study was given orally and in writing. The participation in the study was voluntary. Inclusion criteria for healthy controls were the absence of oral symptoms and/or signs and similar age and gender distribution that the other two study groups.

The patients were administered a questionnaire designed to collect information about possible alterations in taste function. We asked to the patients about their possible alteration in food taste perception Visual analog scales (VAS) were used to score food taste perception (from 0 = no alteration to 100 = extreme alteration).

Burning mouth syndrome was clinically diagnosed on the basis of a history of oral burning and/or oral pain for at least 6 months (2), with no oral signs or evidence of underlying organic causes (primary BMS) (1,2).

Twenty consecutive patients with xerostomia were included, they had dry mouth sensation and a unstimulated salivary flow <0.1 ml/min, determined with the sialometry drainage technique (14).

We excluded patients in all groups with oral lesions, oral infections, abnormal hematological screening findings, patients who used drugs capable of interfering with taste sensation.

-Determination of fungiform papillae density

**Figure 1:**
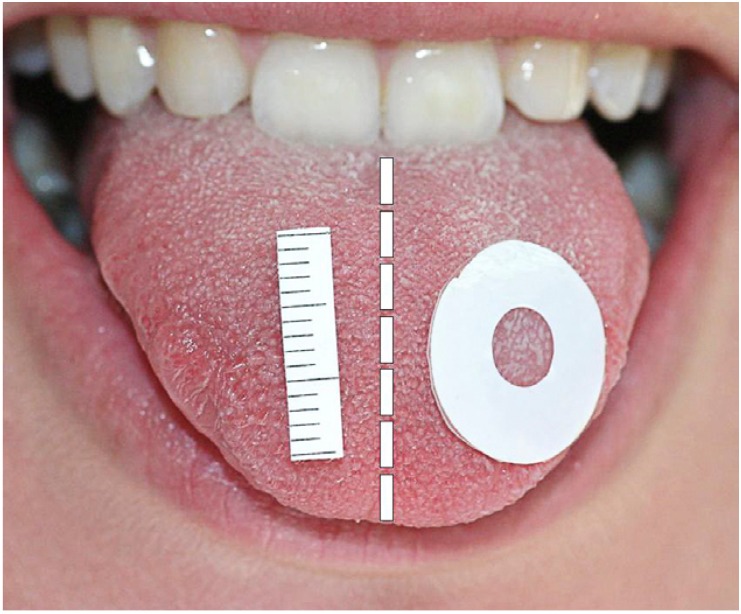
Measurement of fungiform papillae on the dorsal surface of the tongue.

The procedure employed to measure fungiform papillae density was described by Shahbake et al. (15) in 2005. Previously the subjects rinsed their mouth with distilled water. Later, the tongue was dried and a circular filter paper with a central perforation of 6 mm diameter was placed on the tip of the anterior part of the left side of the tongue closest to the midline. A piece of filter paper placed on the right side of the anterior tongue provided a scale to calculate the magnification of each digital image (Fig. 1). Three images were then recorded with a Canon® EOS 300 D camera (Canon® Inc., Vancouver, Canada) using a Tamron® SP AF 90 mm F/2.8 macro-objective (Tamron®, Tokyo, Japan). The digital images were downloaded to a computer and analyzed with Adobe® Photoshop 7.0 (Adobe® Systems Inc., CA, USA). The papillae count was carried out by one same investigator previously blinded to the effects of the study.

-Statistical analysis

Data were analyzed using the SPSS 12.0 statistical package (SPSS® Inc., Chicago, IL, USA). A descriptive study was made of each variable. The Kolmogorov-Smirnov normality test and Levene variance homogeneity test were applied, and the data showing a skewed distribution were analyzed using a nonparametric ranking test. The associations between the different qualitative variables were examined using Pearson’s chi-squared test. We used the Kruskal-Wallis test (for more than two samples) and the Mann-Whitney U-test (for two independent samples) for quantitative variables. Statistical significance was accepted for p£0.05.

## Results

The present prospective study involved 60 subjects with a mean age of 60.24 ± 11.76 years, of with 15 were males (25%) and 45 females (75%). There were no statistically significant differences in relation to age and gender among the three groups ( Table 1).

**Table 1:**
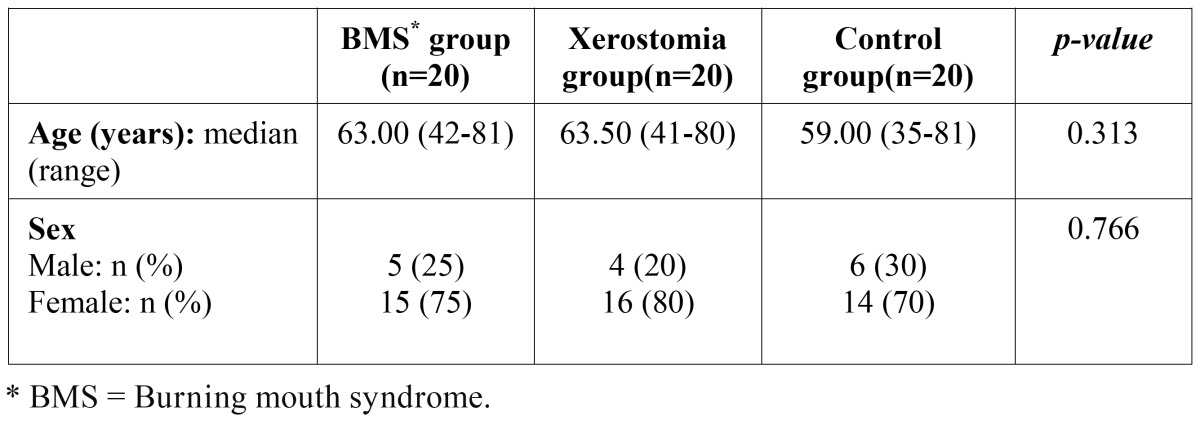
Homogeneity of the study groups in terms of the demographic characteristics (Kruskal-Wallis and Pearson χ2 test).

The patients with BMS showed the greater fungiform papillae density. With statistically significant differences with respect to xerostomia group, though there were no significant differences with respect to the healthy control group ( Table 2).

**Table 2:**
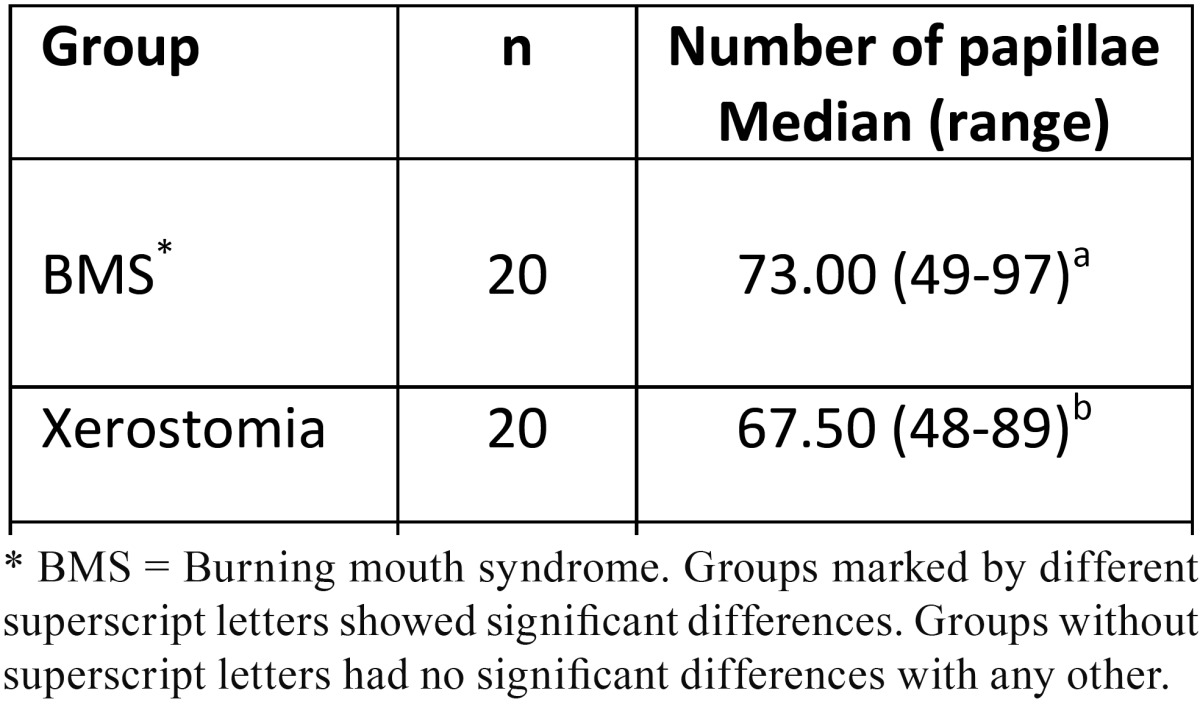
Fungiform papillae density per 19 mm2 (Mann-Whitney U test).

In the BMS group, the number of papillae within the established area of 19 mm2 was 71-90 in 65% of the cases and over 90 in 10%. On the contrary, regarding patients with xerostomia, none of them presented over 90 papillae in the study area and a 70% had ≤70 papillae. Finally, in the control group the number of papillae was over 90 in 15% ( Table 3).

**Table 3:**
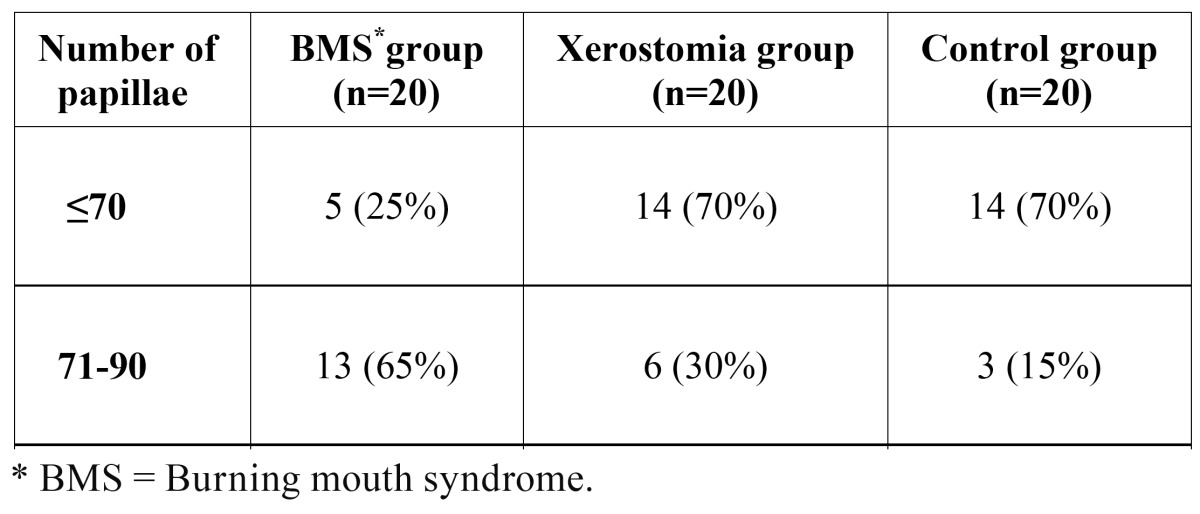
Total number of fungiform papillae located in the study area of 19 mm2.

In relation to score taste alteration as rated by the VAS questionnaire, the patients with BMS and xerostomia had greater food taste alteration and greater difficulty to perceive the flavors (sweet, salty, acid and bitter) than control group, with statistically significant differences ( Table 4).

**Table 4:**
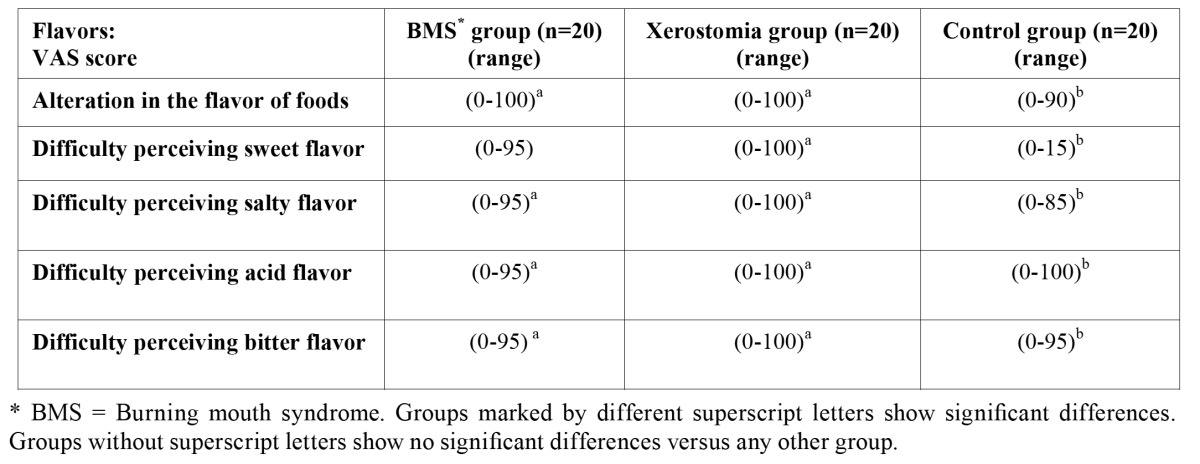
Results of the different groups and taste perception scores (Mann-Whitney U-test).

## Discussion

Our results showed that the patients with BMS had the greater fungiform papillae density.

The taste buds are largely distributed in the epithelium of the dorsal surface of tongue and are less abundant in the mucosa of the palate, pharynx, epiglottis and upper third of the esophagus. In the tongue, the taste buds are located among other in fungiform papillae, in this sense the number of funfiform papillae is directly related to the ability to taste (15-17). We used the digital camera technique to measure fungiform papillae density because it is rapid and easy to carry out; nevertheless, other authors have measured papillae number or density in live videomicroscopy (16). However, although the video microscope is an excellent tool for this purpose, its use is limited to the research laboratory, is a expensive technique and 30-60 minutes are needed to obtain images from an individual of sufficient quality to allow the counting of papillae. This time period is unacceptable to patients with pain (BMS), and is clinically uncomfortable.

Several authors (12,16) arbitrarily chose an area near the tip of the tongue as an indicator of overall fungiform papillae density or of the total number of fungiform papillae, because it is a easily accessible zone. In BMS, patients may experience dysgeusia that increases or decreases upon eating. In some patients this situation represents an alteration in the perception of the intensity of normal food flavors, while others experience a persis-tent and strange taste in the mouth, often of a salty, bitter or metallic nature (2,9,11). In our study the patients with BMS reported alterations in taste perception, with significant differences versus the controls.

Saliva helps to protect taste receptor cells from mecha-nical, thermal, bacterial and viral aggression, and transports taste molecules to taste receptors Saliva is the principal fluid component of the external environment of the taste receptor cells, and as such could play a role in taste sensitivity. In the initial process of taste perception, saliva acts as a solvent for taste substances, and the latter are thus able to diffuse to the taste receptor sites. During this process, some salivary constituents chemically interact with taste substances (17).

Henkin et al. (18) reported a correlation between salivary flow and taste function, while Weiffenbach et al. (19) reported no sig-nificant correlation between the degree of salivary flow deficiency and taste disorders. In the present study, we found that patients with diminished saliva flow showed differences in taste perception with respect to the controls.

Our study has some limitations, since papillae density measurement is a quantitative procedure that should be accompanied by electrogustometry in order to better explore taste sensitivity. In our opinion, measurement of the fungiform papillae may represent a useful and objective tool for the clinical diagnosis of alterations in taste perception. The often disappointing outcome of BMS management may be explained by the still incomplete understanding of its physiopathology. Further research is thus needed in order to clarify the implications between BMS and taste alterations.
